# Frequency of domestic violence in pregnancy and its adverse maternal outcomes among Pakistani women

**DOI:** 10.4314/ahs.v23i4.44

**Published:** 2023-12

**Authors:** Hussain Sana Shahmir, Makhdoom Shafaq, Husain Samia, Hira Aruna, Bano Khadija, Yasmin Haleema

**Affiliations:** 1 Dr Ruth KM Pfau Civil Hospital Karachi; 2 Jinnah Post Graduate Medical Centre; 3 Aziz medical center, obstetrics and gynaecology; 4 Jinnah Post Graduate Medical Centre, department of obstetrics and gynaecology

**Keywords:** Domestic violence, pregnancy outcomes

## Abstract

**Objective:**

To determine the frequency of domestic violence in pregnancy and its adverse maternal outcomes among Pakistani women.

**Material and methods:**

This was a prospective descriptive longitudinal study conducted at the Department of Obstetrics & Gynecology of Jinnah Postgraduate Medical Center, Karachi from October 2021 to March 2022. The questionnaire was filled during an interview. First part included demographic profile, second part comprised of 5 items with ‘yes’ or ‘no’ options. Any positive answer meant woman was subjected to abuse. Adverse maternal outcome was also assessed.

**Results:**

Out of a total of 105 pregnant women, 43(41%) women suffered domestic violence. Verbal or emotional violence (39%) was the most common type of violence. In our study, anemia (71.4%) was the most common complication. Preterm labor (63.8%) was the second on the list. was significantly associated with domestic violence (P-value<0.05). Educational status, employment status, substance abuse, and household monthly income of spouse had a significant association (P-value<0.05) with domestic violence.

**Conclusion:**

Our study shows that high frequency of violence at the time of pregnancy is related to negative maternal outcomes. Women should be screened for violence and support services for such women should be provided in the country.

## Introduction

Domestic violence is defined as any harm, abuse, suffering, coercion, and deprivation, threats that could result in mental, emotional, or physical disability in any individual in public or private. Pregnant females are more prone to domestic abuse. The incidence varies between 1.6% to 66%. 1, 2 According to World Health Organization, 35% of women experience physical or sexual violence from their partners or other individuals in their life. Majority of the violence falls under the partner abuse category. Almost 30% of women report abuse or violent behaviour when in a relationship with their partner. 3 A review that included ninety-two studies from twenty-three different countries claimed that emotional abuse (28%), physical abuse (14%), and sexual abuse (8.0%) was common in pregnancy.^4^ In Pakistan, 44-97% of females suffer lifetime marital abuse and violent behaviour including non-consensual sex or bodily assaults.^1^

Earlier it was thought that pregnancy may play an important role to lessen any kind of abuse. It is still debatable whether pregnancy protects from domestic abuse or is a risk factor to domestic violence. 3, 6 The pattern of physical or emotional abuse during pregnancy varies widely. Some forms of abuse are reported to occur during pregnancy while other abuse is in relation to terminating the pregnancy or occurs after pregnancy has been terminated. Another pattern includes continuous abuse before and during the time of pregnancy. 7 Women have report different types of abuse from their partners which are not exposed due to societal norms, family's reputation, shame, negligence, social stigma, and societal reluctance.^8^ Factors such as lack of family and social support, the low literacy rate in women and unplanned pregnancy are found to be important predictors for domestic abuse against women. 7, 9-11

Domestic abuse during pregnancy increases the risk of homicide and suicide. Significant effects of domestic violence on maternal and fetal outcomes include antepartum haemorrhage, fits, hypertension, preterm labor, emergency caesarean section, low birth weight of the baby, miscarriages, intra-uterine death, maternal and neonatal mortality, and lack of attachment to the children.^1^, ^2^, ^12^, ^13^ Another study showed physical effects like inadequate gain of weight, kidney infections, vaginal and cervical infection, vaginal bleeding, trauma to the abdomen, haemorrhage, aggravation of chronic illnesses, complications during labor, miscarriage, delayed prenatal care, low birth weight, membrane rupture, uterine infection, fractures, hematomas, and death.^14^, ^15^ Intimate partner violence could lead to increased risk of premature birth or low birth weight babies leading to increased risk of neonatal mortality or morbidity.^8^, ^16^ Cultural differences also play an important role. The intercultural study indicated that the imbalance of two genders affects the statistics of violence in the general population.^17^

Domestic abuse is amongst the serious preventable health threats to women. Pregnant women are mostly affected by violence. Still, there is dearth of data on the subject. There are no guidelines for screening domestic abuse in pregnancy in Pakistan. Therefore, the present study focuses on assessing the frequency of domestic violence among pregnant women and its impact on pregnancy outcomes.

## Materials and methods

This “Prospective Descriptive longitudinal Study” was carried out at the Department of Obstetrics & Gynecology of Jinnah Postgraduate Medical Center, Karachi, from October 2021 to March 2022 after taking ethical approval from Institutional Ethical Review Committee. WHO sample size calculator was used. Using the study by James et al. 4 as a reference, with a 95% confidence interval and 6.5% margin of error, the sample size came out to be 105. We, therefore enrolled 105 women. Non-probability consecutive sampling was used. All pregnant women aged 15-49 years, irrespective of the duration of marriage and parity, who came to OPD for registration, antenatal assessment, and those admitted in antenatal ward in the third trimester (28 – 42 weeks of gestation) were enrolled in the study. Those who had a history of assisted conception, psychiatric illness (depression, anxiety disorders, bipolar, Schizophrenia), and language barrier were excluded. These women were interviewed and then followed till delivery. The purpose of the study was explained and written consent was obtained. Participants were assured of privacy and confidentiality. The questionnaire was filled using a direct interview conducted in the local language Urdu by the researchers and retranslated back. Its first part included a demographic profile and the second part comprised of 5 item questionnaires with yes/no options and items included questions on Domestic Violence. For the study purpose, domestic violence was defined as presence of any of the following abuse more than two times.

The abuse was classified as 1) Physical, i.e. assault with weapon/object, slapping pushing, beating hair pulling which resulted in pain or injury; 2) Sexual (forceful sexual intercourse, exhibitionism, exposing to sexually transmitted infections); 3) Verbal/emotional (name-calling, yelling, insulting); 4) Psychological (threatening to harm, silent treatment, socially isolating); or 5) Financial (control over access to economic resources, withholding money and not paying share). A positive response to any of the aforementioned variables signified that woman was subjected to abuse. Intent to pregnancy was evaluated using a single question asking women if this pregnancy was planned. Adverse maternal outcomes were defined as follows; a) preterm labor (labor that begins before 37 weeks of pregnancy),b) antepartum haemorrhage(any amount of bleeding from the genital tract occurring from 24+0 weeks of pregnancy and before the birth of the baby, c) stillbirth ( a baby born with no signs of life at or after 28 weeks gestation), d) caesarean section ( a surgical procedure used to deliver a baby through an incision in the mother's abdomen and uterus), d) anemia ( defined by the WHO as a haemoglobin concentration below 11 g/dL in pregnant women), and e) Maternal Death (defined as the death of a woman while pregnant or within 42 days of termination of pregnancy, irrespective of the duration and site of the pregnancy, from any cause related to or aggravated by the pregnancy or its management, but not from accidental or incidental causes were assessed by taking a history from participants with further confirming by reviewing medical records). Data were entered and analysed by using SPSS 20. Mean and standard deviation were calculated for quantitative variable while frequencies and percentages were calculated for qualitative variables. Factors such as marital status, ethnicity, education, occupation, spousal substance abuse, past medical history and age, socioeconomic status and Intent to pregnancy, etc. were compared with outcomes by using the Chi-Square test was applied by taking P-value < 0.05 as significant.

## Results

A total of 105 pregnant females were included in this study. The mean age of the women was 26.7+/−5.3 years. The mean age of spouse of the patients was 33.4+/−6.01 years. Majority of patients, (61.9%) had been married for less than 5 years, the mean duration of marriage in the study group was 2.5 years. Out of these, 61(58.1%) women had parity of 1-3 and 24(22.9%) had more than 3 children. In this study, most participants were Urdu speaking 45(42.9%). Half of the population was illiterate and only 21(20%) were employed. Intent to Pregnancy was found in 74(70.5%) women. Out of a total of 105 women, 43(41%) women suffered domestic violence. Table 1 summarizes the study population.

**Table 1:** Socio demographic characteristics of study population

Characteristics of study subjects	Frequency (%)
**Age group (in years)**	
≤25 years	49(46.7%)
>25 years	56(53.3%)
**Spouse age (in years)**	
≤35 years	77(73%)
>35 years	28(27%)
**Marriage duration**	
≤5 years	65(61.9%)
>5 years	40(38.1%)
**Parity**	
No Child	20(19%)
1-3 child	61(58.1%)
>3 child	24(22.9%)
**Marital status**	
Married	103(98.1%)
separated	2(1.9%)
**Ethnicity**	
Sindhi	24(22.9%)
Balochi	4(3.8%)
Punjabi	8(7.6%)
Pathan	24(22.9%)
Urdu Speaking	45(42.9%)
**Education**	
None	53(50.5%)
Primary	24(22.9%)
Secondary	24(22.9%)
Intermediate	4(3.8%)
**Last child's gender**	
Male	46(43.8%)
female	59(56.2%)
**Employment status**	
House wife	84(80%)
Employed	21(20%)
**Monthly income in PKR**	
<12,000	25(23.8%)
12,000-15,000	39(37.1%)
>15,000	41(39%)
**Pregnancy intendedness**	
Yes	74(70.5%)
No	31(29.5%)
**Domestic violence**	
Yes	43(41%)
No	62(59%)

### Adverse maternal outcomes

**Figure 1:**
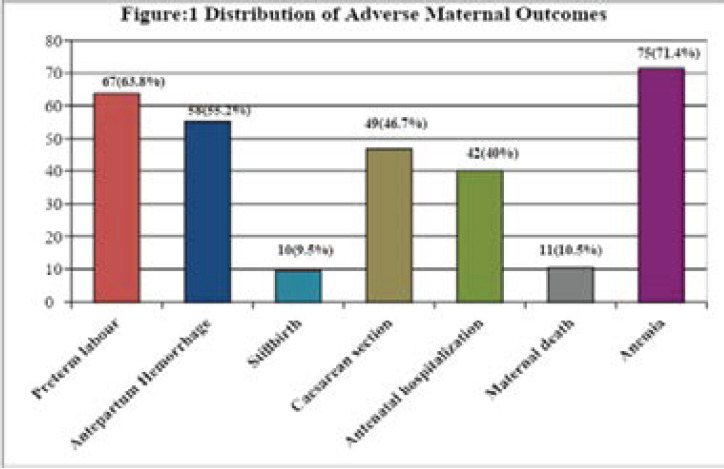
Adverse maternal outcomes

In our study, anemia (71.4%) was the most common complication. Preterm labour (63.8%) and antepartum haemorrhage (55.2%) were other common complications. C/section and antenatal hospitalization were also frequent adverse outcomes. Figure 1

### Domestic violence and its types

**Figure 2:**
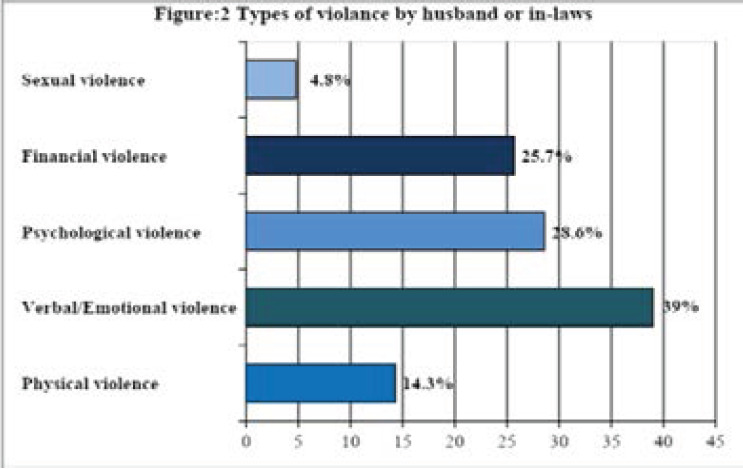
Types of violence by husband or in-laws

Out of a total of 105 women, 43(41%) women were subjected to domestic violence by their husbands or in-laws. Verbal or emotional violence (39%) was the most common type of violence in our population. Figure 2

### Association of maternal factors with domestic violence

There was no significant association of domestic violence with maternal age, duration of the marriage, parity, marital status, ethnicity, educational status. (P-value>0.05) Occupation and intent to fall pregnant were significantly associated with domestic violence (P-value<0.05). Those 32(74.4%) who gave birth to baby girls, were more likely to be subjected to domestic violence. Table 2

**Table 2:** frequency and association of domestic violence according to socio demographic factors of women

Characteristics of study subjects	Domestic violence		P value
	Yes	No	
**Age group (in years)**			0.979
=25 years	20(46.5%)	29(46.8%)	
>25 years	23(53.5%)	33(53.2%)	
Marriage duration			0.059
=5 years	22(51.2%)	43(69.4%)	
>5 years	21(48.8%)	19(30.6%)	
Parity			0.557
No Child	7(16.3%)	13(21%)	
1-3 child	24(55.8%)	37(59.7%)	
>3 child	12(27.9%)	12(19.4%)	
Marital status			0.234
Married	43(100%)	60(96.8%)	
separated	0	2(3.2%)	
Ethnicity			0.233
Sindhi	13(30.2%)	11(17.7%)	
Balochs	36(7%)	1(1.6%)	
Punjabi	2(4.7%)	6(9.7%)	
Pathan	10(23.3%)	14(22.6%)	
Urdu speaking	15(34.9%)	30(48.4%)	
Education			0.143
None	27(62.8%)	26(41.9%)	
Primary	8(18.8%)	16(25.8%)	
secondary	6(14%)	18(29%)	
graduate	2(4.7%)	2(3.2%)	
Last child's gender			0.002*
Male	11(25.6%)	35(46.5%)	
female	32(74.4%)	27(53.5%)	
Employment status			0.022*
House wife	39(90.7%)	45(72.6%)	
Employed	4(9.3%)	17(27.4%)	
Pregnancy intendedness			0.013*
Yes	36(83.7%)	38(61.3%)	
No	7(16.3%)	24(38.7%)	
Maternal outcome			
Preterm Labour	35(81.4) %	32(51.6%)	0.002*
Antepartum haemorrhage	33(76.7%)	25(40.3%)	0.001*
stillbirth	26(60.5%)	23(37.1%)	0.541
caesarean	5(11.6%)	5(8.1%)	0.018*
Antenatal hospitalization	26(60.5%)	16(25.8%)	0.001*
Maternal death	8(18.6%)	3(4.8%)	0.024*
Anemia	37(86%)	38(61.3%)	0.006**

Frequency (%); Chi Square Test was applied.

P-value=0.05 considered as significant.

* Significant at 0.05 level.

### Association of spouse related factors with domestic violence

Domestic violence was also associated with spouse-related factors. There was no significant association of domestic violence with spouse's age and ethnicity (P-value>0.05). While educational status, employment status, substance abuse, and socioeconomic status had a significant association (P-value<0.05) Table 3

**Table 3:** Frequency and association of domestic violence according to socio demographic factors of spouse

Characteristics of study subjects			P value
**Age group in years**			0.811
≤35	31(72.1%)	46(74.2%)	
>35	12(27.9%)	16(25.8%)	
**Ethnicity**			0.623
Sindhi	9(20.9%)	11(17.7%)	
Balochi	5(11.6%)	3(4.8%)	
Punjabi	4(9.3%)	9(14.5%)	
Pathan	12(27.9%)	16(25.8%)	
Urdu Speaking	13(30.2%)	23(37.1%)	
**Education**			0.001*
None	20(46.5%)	3(4.8%)	
Primary	8(18.6%)	4(6.5%)	
Secondary	6(14%)	13(21%)	
Intermediate	9(20.9%)	34(54.8%)	
Graduate	0(0%)	8(12.9%)	
**Occupation**			0.001*
Unemployed	17(39.5%)	2(3.2%)	
employed	17(39.5%)	47(75.8%)	
business	9(20.9%)	13(21%)	
**Substance abuse**			0.001*
None	9(20.9%)	37(59.7%)	
smoking	22(51.2%)	22(35.5%)	
Cocaine	6(14%)	1(1.6%)	
Alcohol	6(14%)	2(3.2%)	
**Monthly income in PKR**			0.007*
<12,000	17(39.5%)	8(12.9%)	
12,000-15,000	12(27.9%)	27(43.5%)	
>15,000	14(32.6%)	27(43.5%)	

Frequency (%); Chi Square Test was applied.

P-value≤0.05 considered as significant.

* Significant at 0.05 level.

## Discussion

Domestic violence is a major indicator of harmful consequences for both mother and the baby. Violence at the time of pregnancy has long-lasting effects. This research was carried out to determine the connection between domestic violence during pregnancy and the harmful pregnancy consequences.

In this report, over 41% of women were subjected to domestic abuse during their pregnancy. The most common form of violence was emotional violence followed by psychological violence. Similar findings were reported by Berhanie et al. In that study, 40.8% of pregnant women were subjected to intimate partner abuse and the most prevalent form of violence was the controlling behaviour of partner and psychological violence. These figures are higher than those reported from Africa 18, Vietnam 19, Iran 20, and WHO multi-country studies 21 where the number of cases of partner abuse identified during pregnancy varied from 23-37%. Data from systematic reviews report that 2–57 % of mothers are assaulted by their spouses during pregnancy 22. Domestic abuse was found to be prevalent in 2% of women in Sweden during their current pregnancy. Longitudinal research throughout developed countries, on the other hand, indicates that domestic abuse during pregnancy is uncommon. Furthermore, there is a paucity of data about possible risk factors related to the occurrence of domestic abuse during pregnancy. 23

The discrepancies in perceived abuse in pregnancy may be attributed to differences in social attitudes, socioeconomic and educational status, methods and scales used for data collection and data analysis, sample size, experiment methodologies, criteria identified. Women's refusal to share the experience of violence in culture is seldom considered. There are certain drawbacks of each sample that can obstruct an effective prevalence rate, such as the sensitivity of the topic, fear of the batterer's retaliation, time-consuming court proceedings, guilt, blame, and potential uncertainty about the results. In contrast to population polls, studies conducted in hospitals or healthcare facilities have a more varied but smaller estimation. Our study showed a higher violence rate.

Jahanfar et al. 24 conducted a study to discover the frequency of domestic abuse and its influence on pregnancy consequences. All the cases of domestic abuse had unplanned births, according to their findings. Women's health care professionals should be mindful of the potentially high incidence of previous or current violence in women seeking abortions. In our study the frequency of women with unplanned births were also subjected to domestic violence.

The results of the present study show no association of age, duration of the marriage, parity, marital status, and ethnicity with domestic violence. However, last-child's gender and occupational status of women showed significant association. Women who had a female child in the last pregnancy and those who were housewives faced more domestic violence (P-value<0.05). Women who were assaulted during their pregnancy had a lower socioeconomic status, had more children, and had a higher rate of unintended pregnancies.

Low household income has proven to be a risk factor in some studies. In our study, 23.8% of the women's spouses earned <12000 Pakistani Rupees/month, 37.1% earned 12000-15000 Pakistani Rupees/month while 39.0% earned < 15000 Pakistani Rupees/month. This positive association could be explained as a reaction to economic challenges faced by low-income families. As they are often subjected to tensions and strains brought on by unemployment and hardship, which has a potential to manifest as hostility and violence.

Low education levels were found to be more prevalent among abused women and/or their male partners than among non-abused women. In our study, women who faced domestic violence had low or no education. It is indeed possible that women with higher levels of education, who are more likely to marry men with higher levels of education, have more capacity for freedom and decision-making authority, especially in family matters, and therefore encounter fewer incidents of domestic assault. The key risk factors for violence against women during this time of their life are alcohol consumption/violence by husbands, low social status of women and their partners, and pregnancy itself. 17 Substance misuse, such as the use of alcohol or cocaine, was shown to be substantially higher among male partners of assaulted women in the sample. Domestic abuse was linked to addiction, husband's supposedly violent nature and the husband's unemployment. 25

Our study also indicated association of adverse maternal outcomes with domestic abuse, which is similar to previous reports.^1^ A meta-analysis of 50 studies has shown increased chances of preterm birth and low birth weight.^26^ Similarly, a recent Egyptian study of 1,857 pregnant women found that violence in pregnancy is linked to a higher rate of negative pregnancy consequences (miscarriage, premature delivery, and premature membrane rupture), and also negative fetal and neonatal consequences (fetal distress, fetal death, and low birth weight).^27^

There was a significant link between preterm birth or low birth weight and exposures to abuse during pregnancy. After controlling for age, educational qualification, occupation, BMI, haemoglobin level, and previous adverse pregnancy consequences, pregnant women who were subjected to abuse in their pregnancy were five times more likely to experience preterm birth (AOR = 5.5; 95 percent CI: 2.1–14.1) and almost six times more likely to give birth to a baby with Low birth weight (AOR = 5.7; 95 percent CI: 2.2–14.9) than those who were not.^19^ Domestic abuse incidence of mothers was shown to be linked to a higher risk of preterm birth. Low Apgar scores were also found to be more common in the children of abused mothers in an earlier study. Perinatal mortality (deaths during the first week of life and fetal deaths or stillbirths) is more likely among women who suffer abuse during pregnancy. 18 Domestic abuse can highly increase the risk of morbidity as well as mortality in mothers and neonates. 28 We found that pregnant women who encountered both anxiety and abuse had a greater chance of delivering a preterm baby and other negative consequences.

There are some limitations in our study. The present study's key limitation is the nonrandomized design of study and it was a specific hospital-based research project. Because of the limited sample size, the findings may not be generalized.

## Conclusion

Our study shows that high frequency of violence at the time of pregnancy is related to negative maternal outcomes. Women should be screened for violence and support services for such women should be provided in the country.
